# Effects of surface materials of self-draining beds on cattle behavior in a temperate climate

**DOI:** 10.5713/ajas.19.0941

**Published:** 2020-02-25

**Authors:** Ping Liu, Lulu Guo, Fulan Zhang, Lin Li, Huaming Mao, Zhaobing Gu

**Affiliations:** 1 Faculty of Animal Science and Technology, Yunnan Agricultural University, Kunming 650201, China

**Keywords:** Cattle, Behavior, Bedding Material, Self-draining Beds

## Abstract

**Objective:**

The objective of the present experiment was to construct self-draining beds to keep surface bedding materials clean and dry for beef cattle comfort in a temperate climate.

**Methods:**

In Experiment 1, a self-draining bed was covered with sand at depths of 10 cm (S-10a), 15 cm (S-15), and 20 cm (S-20) respectively. In Experiment 2, self-draining beds of different sizes were covered with 10 cm of sand (S-10b) and wood shavings (WS) at depths of 15 cm and 20 cm (WS-15 and WS-20). Fifteen cattle were engaged to evaluate the comfort of self-draining beds covered with different bedding materials.

**Results:**

No cattle lay in the feed alley and cattle spent more time lying on S-10a than S-15 or S-20 in Experiment 1 (p<0.01). No difference in lying time was detected between S-15 and S-20 (p>0.05). In Experiment 2, no cattle selected the feed alley as the lying area. Cattle preferred WS-15 as the lying area and time spent lying on WS-20 was slightly higher than on S-10b (p<0.05). Feces weight was higher in the feed alley than in the different bedding areas in both Experiments 1 and 2 (p<0.01).

**Conclusion:**

Sand-bedding depth at 10 cm and WSs at 15 cm above the self-draining bed can provide for the lying comfort of beef cattle. Design of a special feed alley to hold most of the feces to keep bedding materials clean and dry is desirable for organic beef cattle in a loose barn.

## INTRODUCTION

Production of beef cattle is a major agricultural industry and significant part of the rural economy in Yunnan Province, China. Great technological advancements in beef cattle production have been achieved during the past few decades, but profitability is still low in most indoor beef cattle operations. To improve this situation and satisfy the demand for higher quality beef, the Yunnan Government issued a policy for the organic beef cattle industry in 2018. Though a series of standards have been set, organic beef production faces great challenges regarding improvement in animal housing conditions.

Animal-friendly flooring is a basic requirement for organic beef production, and floor type significantly affects production performance and animal welfare. Concrete is widely used as flooring material in dairy farms as this is affordable and convenient to manage. However, bulls kept on concrete floors display uncomfortable lying behavior and less lying time than bulls kept in a bedded lying area [1]. Fully slatted concrete floors are used in loose housing systems in many European countries for efficient management (e.g. ease of manure handling, cleanliness of the pen and hence increased animal hygiene) but steers on slatted floors also display uncomfortable lying behavior, such as more frequent posture changes, than on a solid floor [2]. Organic beef cattle production regulations do not permit slatted floors to be used for beef cattle. Cattle must be provided with bedding materials in a loose housing system, since these affect the health and welfare of the cattle [3]. Cattle ruminate for a long time when lying comfortably on soft bedding materials.

Straw and other crop residues have been used as cushion ing materials to improve the lying comfort of cattle. However, the use of straw, saw dust and wood shavings (WS) as bedding materials on a solid floor for large numbers of cattle is impractical due to the production of large quantities of urine and the seasonal shortage of bedding materials [4,5]. To reduce the amount of straw required, mattresses and rubber mats have been used as alternative bedding materials for dairy cows in free stalls [6]. Mattresses and rubber mats with high thermal resistance may be unsuitable for use during hot summers [7] and are inappropriately used as bedding materials for low self-draining capacity when cattle are housed in loose barns with common lying areas [8].

Previous research has predominantly focused on the effects of bedding materials on behavior of dairy cows while knowledge of similar effects on behavior of beef cattle is scarce due to economic constraints and short raising time. Keeping bedding material dry is challenging because there may be seasonal shortages of appropriate material that lead to untimely supplementation or replacement. Poor quality organic bedding may cause reproductive disorders in cattle due to the presence of bacteria and mycotoxins that can be transferred to the animals [9,10].

**Figure 1:**
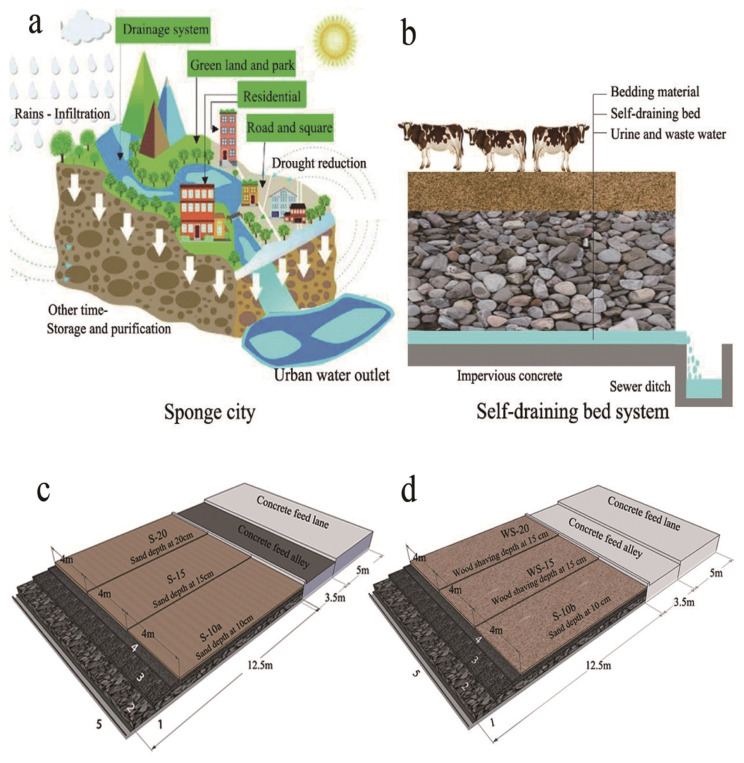
Sponge city concept and self-draining bed system for beef cattle. (a) sponge city concept; (b) self-draining bed system; (c) Pen dimensions, and self-draining bed covered with sand at different depths in Experiment 1; (d) self-draining bed covered with sand and/or wood shaving at different depths in Experiment 2. 1, impervious concrete layer; 2, rubble stone layer at depth of 30 cm; 3, cobblestone layer at depth of 20 cm; 4- gravel layer at depth of 10 cm; 5, sewer ditch.

Inspired by a product made by Sponge city (Figure 1a) designed to passively absorb rainfall to address problems caused by drought and flood disasters [11], we constructed self-draining beds that maintain bedding surface quality without polluting ground water and soil (Figure 1b). Self-draining beds could increase the service life of bedding materials. Self-draining beds that farmers covered with available bedding materials were evaluated in terms of cattle behavior. The distribution of feces in different parts of loose housing barns with feed alleys combined with self-draining beds were also evaluated to provide guidance for bedding material management. The objective of the present experiment was to construct self-draining beds to keep surface bedding materials clean and dry for beef cattle comfort in a temperate climate.

## MATERIALS AND METHODS

### Self-draining bed system construction

A feed alley in front of the feed bunk was 3.5 m wide. A pen with a self-draining bed serving as the lying area was 12.5 m long and 12.0 m wide. The lowermost layer of the self-draining bed consisted of impervious concrete and had a 2% slope and the middle layer, 30 cm deep rubble stone. The top layer consisted of 20 cm deep cobblestone and 10 cm deep gravel. The self-draining bed was divided into three areas (12.5 m× 4.0 m) demarcated with round-timber and cattle had access to all three areas in Experiment 1. The three areas of the self-draining bed were covered with sand (2 mm screen) at depths of 10 cm (S-10a), 15 cm (S-15), and 20 cm (S-20), respectively (Figure 1c). In Experiment 2, another self-draining bed with the same dimensions as that in Experiment 1 was similarly divided into three areas (12.5 m×4.0 m) using round-timber and cattle had access to all three areas in Experiment 2. The three areas of the self-draining bed were covered with 10 cm deep sand (S-10b), and WS at depths of 15 cm (WS-15) and 20 cm (WS-20) (Figure 1d). WS were 20 to 40 mm long. Sand and wood shavings were groomed manually and supplied timeously to maintain a constant depth in both Experiments 1 and 2.

### Animal management

The study was conducted in Dehong State, China (24.43ºN, 98.57ºE and 870 m above sea level) in December 2018 and animal handling procedures were approved by the Animal Ethics Committee under the Yunnan Province Animal Welfare Act China (20071001). Fifteen healthy female Simmental cattle with similar body condition scores were engaged in the study, and each bedding area (50 m^2^) could meet the lying requirements for all animals. Cattle were fed the whole corn silage and concentrate diet and had free access to fresh drinking water.

### Animal behavior observation

Behavior of the 15 cattle housed on self-draining bed was observed using continuous sampling with digital camera for four consecutive days (96 hours) to detect the behavioral preference at different sand bedding areas in Experiments 1. The same 15 cattle were transferred to another self-draining bed to detect the behavioral preference at different bedding areas covered with sand and wood shavings using continuous sampling for four consecutive days (96 hours). Cattle behavior included standing and lying down. Animals were considered to be lying down when their body trunk was in contact with the ground in the feed alley or on bedding materials regardless of posture. Cattle were considered standing when their body weight was supported with four legs. After completing the field trail, all cattle behavioral recordings were observed continuously by the same observers.

### Comfort index evaluation

A comfort index (CI) was used to evaluate the lying comfort of different bedding materials. CI is calculated by dividing the number of cattle lying on the bedding surface by the total number of cattle [12,13]. The number of cattle lying on the bedding area was recorded at 10:00, 16:00, and 20:00 to calculate CI. Feces on the feed alley floor and different bedding surfaces were collected and weighed twice daily.

### Statistical analysis

Analyzes were performed using IBM SPSS 21.0 software (IBM Corporation, New York, USA). Behavioral parameters (lying and standing at special area) were obtained from individual cattle. Standing and lying time, and weight of feces were analyzed using one-way analysis of variance following a Shapiro-Wilk analysis. The Kruskal-Wallis test was applied to analyze the effect of the self-draining bed covered with different bedding materials on behavioral frequencies [14].

## RESULTS

### Behavioral parameters in Experiment 1

Cattle behavioral time allocation and frequencies in Experiment 1 are shown in Tables 1 and 2, respectively. Significant differences were detected in time spent standing for cattle in the feed alley at S-10a, S-15, and S-20. Cattle spent more time standing (including walking and feeding) in the feed alley than S-10a, S-15, or S-20 (p<0.01). More time was spent standing at S-10a than S-15 or S-20 (p<0.01) but no significant difference was detected between S-15 and S-20 (p>0.05). Feeding is synchronized with standing for healthy cattle. When calculating feeding time, cattle still spent more standing time in the feed alley (p<0.01). Cattle spent no time lying in the feed alley and 12.3 hours per day lying down on the three sand bedding material areas. Unexpectedly, cattle spent more time lying in S-10a than S-15 or S-20 (p<0.01) but no difference in lying time was detected between S-15 and S-20 (p>0.05). No differences in behavioral frequency were detected between the different areas in Experiment 1 (p>0.05) but cattle in S-10a showed higher standing and lying frequencies than those in S-15 and S-20.

### Behavioral parameters in Experiment 2

Cattle behavioral time allocation and frequencies in Experiment 2 are shown in Tables 3 and 4, respectively. Cattle spent more time standing in the feed alley than S-10b, WS-15, or WS-20 (p<0.05). When calculating feeding time, cattle also spent more time standing in the feed alley than the S-10b, WS-15, or WS-20 (p<0.05). Cattle did not lay down in the feed alley when provided with sand and wood shavings as bedding materials, and cattle spent 13.9 hours lying down on the three sand bedding material areas per day. Cattle preferred WS-15 and slightly more time was spent lying down in WS-20 than S-10b (p<0.05). No significant differences were detected in behavioral frequency at different areas in Experiment 2 (p>0.05) but cattle at WS-15 had higher standing and lying frequencies compared with those at S-10b and WS-20.

### Cattle lying comfort index

The cattle lying CI is summarized in Table 5. When comparing the CI of cattle housed in a loose barn with access to a self-draining bed covered with different depths of sand, a higher CI was obtained at S-10a than at S-15 or S-20 (p<0.05) but no difference was detected between S-15 and S-20 in Experiment 1. In Experiment 2, cattle showed higher CI in WS-15 and WS-20 than S-10b (p>0.05) when provided with sand and wood shavings above the self-draining bed at the same time, but no difference was detected between the CI at WS-15 and WS-20 (p>0.05).

### Distribution of cattle feces

Table 6 shows the weight of cattle feces at different areas. The weight of cattle feces was higher in the feed alley floor than S-10a, S-15, and S-20 in Experiment 1 and the result was the same in Experiment 2. The weight of the feces in the feed alley was higher than that in S-10b, WS-15, and WS-20 (p<0.01) but no significant difference was detected in distribution of feces in the three bedding areas (p>0.05).

## DISCUSSION

Cattle lie down to ruminate and sleep for approximately 12 to 15 hours per day in a comfortable environment [15,16]. Cattle that are tied up are usually raised on a solid hard floor that negatively affects lying time and feeding utilization efficiency. According to production standards, organic beef cattle must be loose-housed with clean, dry bedding materials. Bedding materials are often soiled with urine and feces on a solid floor. A slatted floor can increase the cleanliness of the skin and sanitary conditions of the bedding surface [17], but beef cattle kept on a slatted floor have a high incidence of injuries [18]. Rubber mats are unsuitable for use as bedding materials because these cause cattle to fall and decreases their levels of cleanliness [19], unless combined with other soft, grained materials that increase their surface friction.

Lying time plays an important role in increasing bouts of rumination and feed efficiency of beef cattle, both of which depend significantly on bedding base and materials. Lying preference is an effective indicator of the lying CI for cattle. Time spent lying on a thin layer of sand bedding (S-10a) was unexpectedly longer than for both S-15 and S-20 in Experiment 1, contrary to previous results showing that cows preferred more sand bedding in stalls [20]. Bickert et al [21] considered that the depth of bedding materials needs to be at least 15 cm above a cement base to maintain lying comfort for cattle. A possible reason for our findings is that the sand-gravel cushion beneath bedding materials (10 cm sand) improved the level of lying comfort for cattle in our study. Cattle feces were weighed twice daily to analyze the distribution of feces, with the aim of keeping the lying material surfaces clean and dry. Due to the characteristics of large fluidity, the decreased depth of sand bedding with use reduced the lying time of cattle [21]. Sand bedding at a constant depth of S-10a above a self-draining bed may maintain lying comfort for cattle. Sand depth above a self-draining bed may affect the behavioral transition from lying to standing. Deep sand bedding may not be greatly used by cattle for lying in a loose barn, when sand is maintained at a constant depth.

After transferring to another similar self-draining bed covered with sand bedding at 10 cm deep (S-10b) and WSs at 15 cm (WS-15) and 20 cm (WS-20) deep in Experiment 2, cattle were found to prefer WS-15 and WS-20 to S-10b. The difference in lying preference in Experiments 1 and 2 indicated that the lying CI of WSs is greater than that of sand in a temperate climate and this is consistent with previous studies [22]. The difference in thermal conductivity between sand and wood shavings may affect thermal comfort during lying and hence usage of the lying area [23,24]. In addition, WSs may have a greater bearing capacity for cattle’s feet and body weight than sand. The good physical and thermal comfort of WS may explain the high lying preference of cattle in a temperate climate.

CI as a critical factor in production is widely used to eval uate the quality of the environment of dairy cows and similar criteria were used to evaluate the lying comfort of cattle and quality of bedding materials. Lying is a high priority behavioral requirement for cattle [25]. CI was consistent with the time budget of lying in Experiments 1 and 2. Self-draining beds keep the bedding materials dry and clean, increasing the lying comfort. High CI on a thin layer of sand (S-10a) may indicate that this can provide comfort area for lying and kneeling (posture transition from lying to standing or vice versa). WS had a higher CI, indicating that WS at 15 or 20 cm deep can provide physical and thermal comfort in a temperate climate. WS with good bearing ability facilitate cattle lying down or standing. Self-draining beds covered with bedding materials serve dual functions as loafing and lying areas for cattle, thus keeping bedding materials dry and clean, and hence providing a high lying CI.

Cattle skin cleanliness is an important parameter for cattle health, thermoregulation, reproductive performance and meat hygiene [26,27], but skin is often soiled with feces and urine. Cattle feces are spread over a large area and the distribution is closely associated with skin cleanliness. Cattle often defecate while walking, standing and getting up [28] and the distribution of feces was closely associated with standing time spent in the feed alley floor in both Experiments 1 and 2. High standing frequencies were found in bedding areas S-10a, S-15, and WS-15 but the weights of the feces in the three bedding areas were comparatively lower than those in the feed alley. This can be explained by the fact that no cattle defecated while in the lying posture in Experiments 1 and 2. Robichaud et al [29] also reported only a small portion of cattle defecating in the lying posture.

## CONCLUSION

Self-draining beds covered with sand at a constant depth of 10 cm or WS at a depth of 15 cm can provide beef cattle with comfortable lying surfaces. In our experiments, no cattle selected the hard concrete alley floor as a lying area when provided with comfortable bedding materials. Designing a special feed alley to hold most of the feces to keep bedding materials clean and dry for organic beef cattle in a loose barn is desirable.

## Figures and Tables

**Table 1:** Time allocation (min) in the feed alley and self-draining bed covered with sand at different depths (means±standard error) for 24 h in Experiment 1

Location	All standing	Standing only	Lying
Feed alley	455.1±16.3^a^	228.9±17.2^a^	-
S-10a1)	121.9±19.2^b^	121.9±19.2^b^	336.5±51.3^a^
S-151)	49.0±6.1^c^	49.0±6.1^c^	198.8±40.8^b^
S-201)	71.3±13.2^c^	71.3±13.3^c^	203.0±52.2^b^

1) S-10a, a self-draining bed was covered with sand at depths of 10 cm; S-15, a self-draining bed was covered with sand at depths of 15 cm; S-20, a self-draining bed was covered with sand at depths of 20 cm.

a,b Means within variable having different superscript letters differ (p<0.05).

**Table 2:** Behavioral frequencies in the feed alley and self-draining bed covered with sand at different depths for 24 h in Experiment 1 (means±standard error)

Location	Standing	Standing only	Lying
Feed alley	10.16±1.07	10.16±1.07	-
S-10a1)	14.59±3.08	14.59±3.08	6.29±1.63
S-151)	14.22±1.57	14.22±1.57	2.89±0.50
S-201)	9.94±1.47	9.94±1.47	3.11±1.46
p-value	0.12	0.12	0.16

1) S-10a, a self-draining bed was covered with sand at depths of 10 cm; S-15, a self-draining bed was covered with sand at depths of 15 cm; S-20, a self-draining bed was covered with sand at depths of 20 cm.

**Table 3:** Time allocation (min) in the feed alley and self-draining bed covered with sand and wood shavings at different depths for 24 h in Experiment 2 (mean±standard error)

Location	All standing	Standing only	Lying
Feed alley	410.1±9.4^a^	119.2±11.8^a^	-
S-10b1)	66.3±8.3^bc^	66.29±8.3^bc^	245.6±43.3^a^
WS-151)	86.8±14.3^b^	86.8±14.3^b^	323.1±38.5^b^
WS-201)	42.6±5.6^c^	42.6±5.6^c^	264.4±42.7^a^

1) S-10b, a self-draining bed was covered with sand at depths of 10 cm; WS-15, a self-draining bed was covered with wood shavings at depths of 15 cm; WS-20, a self-draining bed was covered with wood shavings at depths of 20 cm.

a–c Means within variable having different superscript letters differ (p<0.05).

**Table 4:** Behavioral frequencies in the feed alley and self-draining bed covered with sand at different depths for 24 h in Experiment 2 (means±standard error)

Location	Standing	Standing only	Lying
Feed alley	8.32±1.01	8.25±0.94	-
S-10b1)	8.21±1.72	8.21±1.72	2.54±0.88
WS-151)	11.96±1.79	11.96±1.79	3.61±0.85
WS-201)	6.57±0.55	6.57±0.55	2.79±0.60
p-value	0.13	0.13	0.71

1) S-10b, a self-draining bed was covered with sand at depths of 10 cm; WS-15, a self-draining bed was covered with wood shavings at depths of 15 cm; WS-20, a self-draining bed was covered with wood shavings at depths of 20 cm.

**Table 5:** Comfort index on the self-draining beds covered with sand or wood shavings

Groups	CI
Experiment 1	
S-10a1)	37.78±2.22^a^
S-151)	15.72±2.46^b^
S-201)	16.67±2.89^b^
Experiment 2	
S-10b2)	13.33±3.55^a^
WS-152)	27.50±3.72^b^
WS-202)	25.83±4.84^b^

CI, comfort index.

1) S-10a, a self-draining bed was covered with sand at depths of 10 cm; S-15, a self-draining bed was covered with sand at depths of 15 cm; S-20, a self-draining bed was covered with sand at depths of 20 cm.

2) S-10b, a self-draining bed was covered with sand at depths of 10 cm; WS-15, a self-draining bed was covered with wood shavings at depths of 15 cm; WS-20, a self-draining bed was covered with wood shavings at depths of 20 cm.

a,b Means within variable having different superscript letters differ (p<0.05).

**Table 6:** Daily cattle feces weight (kg) at different areas (mean±standard error)

Defecation area	Feces weight
Experiment 1	
Feed alley	89.3±9.4^A^
S-10a1)	44.6±8.3^B^
S-151)	39.3±6.8^B^
S-201)	38.9±9.3^B^
Experiment 2	
Feed alley	63.2±7.3^A^
S-10b2)	37.5±6.9^B^
WS-152)	31.1±2.1^B^
WS-202)	27.7±2.4^B^

1) S-10a, a self-draining bed was covered with sand at depths of 10 cm; S-15, a self-draining bed was covered with sand at depths of 15 cm; S-20, a self-draining bed was covered with sand at depths of 20 cm.

2) S-10b, a self-draining bed was covered with sand at depths of 10 cm; WS-15, a self-draining bed was covered with wood shavings at depths of 15 cm; WS-20, a self-draining bed was covered with wood shavings at depths of 20 cm.

A,B Means within variable having different superscript letters differ (p<0.01).
